# Enrichment of G4DNA and a Large Inverted Repeat Coincide in the Mitochondrial Genomes of *Termitomyces*

**DOI:** 10.1093/gbe/evz122

**Published:** 2019-06-18

**Authors:** Mathijs Nieuwenhuis, Lennart J J van de Peppel, Freek T Bakker, Bas J Zwaan, Duur K Aanen

**Affiliations:** 1 Laboratory of Genetics, Wageningen University & Research, The Netherlands; 2 Biosystematics Group, Wageningen University & Research, The Netherlands

**Keywords:** fungi, mtDNA, G-quadruplex, inverted repeat, Lyophyllaceae

## Abstract

Mitochondria retain their own genome, a hallmark of their bacterial ancestry. Mitochondrial genomes (mtDNA) are highly diverse in size, shape, and structure, despite their conserved function across most eukaryotes. Exploring extreme cases of mtDNA architecture can yield important information on fundamental aspects of genome biology. We discovered that the mitochondrial genomes of a basidiomycete fungus (*Termitomyces* spp.) contain an inverted repeat (IR), a duplicated region half the size of the complete genome. In addition, we found an abundance of sequences capable of forming G-quadruplexes (G4DNA); structures that can disrupt the double helical formation of DNA. G4DNA is implicated in replication fork stalling, double-stranded breaks, altered gene expression, recombination, and other effects. To determine whether this occurrence of IR and G4DNA was correlated within the genus *Termitomyces*, we reconstructed the mitochondrial genomes of 11 additional species including representatives of several closely related genera. We show that the mtDNA of all sampled species of *Termitomyces* and its sister group, represented by the species *Tephrocybe rancida* and *Blastosporella zonata*, are characterized by a large IR and enrichment of G4DNA. To determine whether high mitochondrial G4DNA content is common in fungi, we conducted the first broad survey of G4DNA content in fungal mtDNA, revealing it to be a highly variable trait. The results of this study provide important direction for future research on the function and evolution of G4DNA and organellar IRs.

## Introduction

Mitochondria are a vital component of virtually all eukaryotic life, generating energy in the form of ATP and regulating the respiratory metabolism. Mitochondria maintain their own genome, a legacy of their endosymbiotic origin. However, despite their common ancestry and conserved function, mitochondrial genomes (mtDNA) of major lineages such as plants, fungi, and metazoans differ considerably not only in size, content, and structure but also in mutability, inheritance, and replication (Birky 2001; Burger et al. 2003; Lynch et al. 2006; Wilson and Xu 2012; Smith and Keeling 2015). Fungi by themselves contain a huge variety of mtDNA shapes and sizes, and are an opportune group for studying the causes and effects of mtDNA diversity.

Like the vast majority of eukaryotes, in most fungi mtDNA transmission during sexual reproduction is uniparental. Species of the genus *Termitomyces* (Lyophyllaceae, Basidiomycota), a clade of obligate mutualistic symbionts of termites, are an exception. During mating in *Termitomyces*, nuclei of both parents enter a shared cytoplasm from which offspring cells grow, rather than one parent adopting a nucleus from the other as is typical for most basidiomycetes (Nobre et al. 2014). This implies that fertilized cells initially are heteroplasmic (containing the mitochondria of more than one parent), increasing the potential for recombination of mtDNA. This is similar to what has been demonstrated in *Agaricus bisporus* (Xu et al. 2013), another fungus that reproduces in this fashion. These deviations from “canonical,” uniparentally inherited, nonrecombining mtDNA render these fungi of high interest for studies on mtDNA evolution, as they enable testing hypotheses on possible influence of recombination on mtDNA selection and evolution. However, to date, no mtDNA sequence for *Termitomyces* or any closely related fungus has been published. In this article, we reveal the mtDNA sequences of seven species of *Termitomyces*, showing they have a mitochondrial inverted repeat (IR) that generally occupies half of the genome, duplicating several key genes and increasing the mtDNA size significantly. In addition, we found an abundance of G-quadruplex (G4DNA) motifs, prompting the question whether these two structural features are functionally correlated. We also sequenced the mtDNA of five other Lyophyllaceae: *Blastosporella zonata*, *Tephrocybe rancida*, *Myochromella boudieri*, *Asterophora parasitica*, and *Tricholomella constricta*, to estimate the origin of the IR and the G4DNA increase.

G-quadruplex DNA, or G4DNA, is a naturally occurring conformation of DNA that folds in a four-stranded conformation rather than the common double helix. Although originally considered as an in vitro curiosity, G4DNA is now known to exist in living cells of ciliates, humans, yeasts, nematodes, and others (Schaffitzel et al. 2001; Johnson et al. 2008; Verma et al. 2009; Koole et al. 2014). The formation of G4DNA requires between one and four strands of DNA containing tandemly repeated guanine motifs. The shape and stability of G4DNA depends on the number of comprising strands and their nucleotide sequence. G4DNA complexes can be stabilized or destabilized by specific binding proteins, particularly helicases (Mendoza et al. 2016).

G4DNA can have many effects depending on the location at which they occur and the shape they form. Effects associated with G4DNA include: replication fork stalling, altered gene expression, recombination, double-stranded break (DSB) formation, and genome instability (Maizels 2006; Rawal et al. 2006; Verma et al. 2009; Bharti et al. 2014; Huang et al. 2015; Cui et al. 2016; Rawal et al. 2006). In addition, the telomeres of many eukaryotes comprise G4DNA forming motifs. G4DNA formation at the ends of telomeres regulates telomerase activity (Zaug et al. 2005). G4DNA is also enriched around replication origins in vertebrates, and is considered to play a role in genome replication (Paeschke et al. 2011; Valton et al. 2014). G4DNA forms more frequently in cancer cells (Biffi et al. 2014), and G4DNA motifs are prevalent in promotors of oncogenes (Eddy and Maizels 2006). Given these properties, most research on G4DNA focuses on its potential role in cancer development (Hänsel-Hertsch et al. 2017).

Recent studies have employed predictive algorithms to map potential G4DNA in sequenced genomes based on sequence patterns (Eddy and Maizels 2006; Rawal et al. 2006; Du et al. 2008; Yadav et al. 2008; Capra et al. 2010; Garg et al. 2016). This revealed a plethora of G4DNA motifs in human, eumetazoan, plant, and yeast DNA. In human mitochondria, G4DNA motifs were found in close proximity to regions of instability related to mitochondrial dysfunction and diseases (Bharti et al. 2014). Capra et al. (2010) analyzed the G4DNA motif content of the nuclear and mitochondrial genome of *Saccharomyces cerevisiae*, and found an ∼10-fold increase of G4DNA motif content in mtDNA compared with nuclear DNA. However, a broad, comparative survey of G4DNA content of fungal nuclear and mtDNA is still lacking.

Large inverted duplications are a common, ancestral feature in chloroplast genomes (Palmer 1985; Turmel et al. 1999). They generally drive a process of continuous homologous recombination (HR) which maintains sequence equality between the two repeat copies, and inverts the orientation of the nonduplicated regions (Palmer 1983; Aldrich et al. 1985). IRs are comparatively rare in mtDNA, being a derived character opposed to the chloroplast IR, although mitochondrial IRs are common in several stramenopile lineages (Hudspeth et al. 1983; Brien et al. 2014). The function of large IRs in organelle genomes is unclear. In chloroplasts, they sometimes appear to promote genomic stability by reducing mutation rates (Palmer and Thompson 1982; Maier et al. 1995), but in other studies this effect was not observed (Blazier et al. 2016). HR-driven repair of DSBs within the IR using the alternate copy as a template is thought to contribute to the reduced mutation rate observed in previously mentioned studies (Gualberto et al. 2014; Zhu et al. 2016). The importance of HR-mediated repair of DSBs is well established in many organisms (Shrivastav et al. 2008). In *Candida albicans*, the mitochondrial IR is centered on a replication origin, and appears to regulate genome replication through recombination-driven replication with the IR as an initiation site (Gerhold et al. 2010).

In this article, we present new mitochondrial genome sequences of 12 basidiomycete species, and explore the occurrence of the IR and G4DNA motifs in these genomes. Given that both structural phenomena have been associated with genome replication and (in)stability, we hypothesize that the IR is correlated with enriched G4DNA in *Termitomyces*, and that selection for increased genomic stability may have driven expansion of the IR in these species.

## Materials and Methods

### DNA Material and Sequencing

We selected species for this study based on their phylogenetic position (Hofstetter et al. 2014) and ecology. *Termitomyces* strains were selected to include at least two strains for each of the three most species-rich associated termite host genera (*Macrotermes*: T132, T123, DKA19; *Microtermes*: Mi166, T13; *Odontotermes*: T32, T159) (Aanen et al. 2002). *Termitomyces* strains associated with these genera cover the phylogenetic diversity of this fungal genus. *Blastosporella zonata* and *T.rancida* were selected as representatives of the sister clade of *Termitomyces*. *Myochromella boudieri* was selected as representative of the sister clade to the previous two clades. Finally, we selected *Tricholomella constricta* and *Ast.parasitica* as representatives of more distantly related Lyophyllaceae. Material used for DNA isolation was obtained from pure cultures. Fungal isolates were grown on malt yeast extract agar (MYA; per liter demi water: 20 g malt extract, 2 g yeast extract, 15 g agar). The *Termitomyces* isolates were grown at 25 °C in the dark. *Tricholomella constricta*, *Ast.parasitica*, *T.rancida*, and *M. boudieri* isolates were grown at 15 °C in the dark. For *Termitomyces* sp. DKA19, no culture was present and hyphal nodules collected from a termite mound were used instead. Nodules were stored in pure ethanol at −20 °C. Prior to extraction, five nodules were rinsed with clean pure ethanol and dried on filter paper.

DNA was extracted using a cetyltrimethylammonium bromide (CTAB) protocol (supplementary data 1, Supplementary Material online). Library preparation and whole-genome sequencing was performed by Novogene (Hong Kong) using the Illumina Hiseq 2500 platform. Generated reads were 150 bp long and the insert size was 500 bp. Because of the high coverage of mtDNA, we generally used a subsample of one to two million reads to assemble the mitochondrial genomes. This speeds up the assembly process and limits interference of nuclear DNA.

### Sequence Assembly

To reconstruct mitochondrial genome sequences from whole-genome sequencing data, we took a reference-based iterative read baiting approach using the IOGA-pipeline (Iterative Organelle Genome Assembly; Bakker et al. 2016). IOGA performs quality filtering and adapter trimming of reads using BBduk, reference mapping using BBmap, and read assembly using both SOAPdenovo2 (Lam et al. 2013) and SPAdes (v3.1.1; Bankevich et al. 2012). Resulting assemblies are evaluated using maximum likelihood with the program ALE (Clark et al. 2013) to assist in identifying the “best” assembly. As IOGA-assemblies did not always capture the full mitochondrial genome sequence in one contig, subsequent scaffolding of contigs was performed if necessary using SSPACE and GapFiller (Boetzer et al. 2011). Final assembly of remaining contigs was performed manually to resolve the IR as follows: 1) contigs covering the IR were identified by a 2-fold increase in sequence coverage; 2) IR-SC boundaries were identified by 95-bp sequence overlaps between the IR and SC contigs; 3) such contigs were joined and the overlapping sequence was merged; 4) reads were mapped back to the resulting scaffold to check whether paired reads surrounding the IR-SC boundary mapped correctly. Some gaps in the assembly arose from sequence artifacts due to long (10 bp+) stretches of monomer repeats. Reads covering these monomers would accumulate sequence errors downstream of the repeat, resulting in termination of the contig. We resolved these gaps by removing the sequence between the gap and the repeat, leaving roughly ten bases of the monomeric repeat intact. We then mapped reads back to the repeat to roughly estimate its length and check if read pairs mapped correctly to either side of the repeat. We identified potential assembly errors by mapping reads back to the draft assembly and inspecting the SAM-file for regions with reduced coverage, SNPs, or incorrect read pairings.

### Annotation

Initial annotation of the first mitochondrial genome (T132) was done manually by performing a BLAST search of the genome against closely related genomes such as *Tricholoma matsutake* and *Pleurotus ostreatus* in the NCBI database. Subsequent annotations used the annotated T132 genome as a reference, by transferring annotations from T132 with a 70% sequence similarity threshold in Geneious v11 (Kearse et al. 2012), and then manually adjusting as needed. Exon boundaries were approximated by comparing amino-acid sequences to references with BlastP. Additional (nonconserved) ORFs were identified with Geneious. We used RNAWeasel (Lang et al. 2007) to identify tRNAs and the small ribosomal subunit. Transcriptomics data (SRA accession SRR5944782) were available for one *Termitomyces* strain of the same suspected species as *Termitomyces* sp. T132 (da Costa et al. 2018), which we used to check our annotation. We aligned RNAseq reads to the annotated genome using TopHat (Trapnell et al. 2009) with Bowtie2 (Langmead and Salzberg 2012) using the sensitive parameter and otherwise default settings. Although for some genes read coverage was limited, for most genes, we confirmed the intron–exon boundaries in our annotation. Images of the annotated genomes were produced using OGDRAW (Lohse et al. 2013).

### PCR Confirmation of IR Borders

To confirm the presence of the mitochondrial IR in vitro, a touchdown PCR was conducted. The primers that were used were designed manually based on the mitochondrial genome assembly (supplementary data 2*A*, Supplementary Material online). We designed primers to cover all four expected border regions between the IR, SC1, and SC2 for two *Termitomyces* species: T132 and T13. The master mix and PCR program used can be found in the supplementary data (2*B*), Supplementary Material online. The PCR products were sequenced using the forward primer at Eurofins Genomics (Ebersberg, Germany).

### Phylogenetic Analysis of Fungal mtDNA

We reconstructed two phylogenetic trees: one focusing on the Lyophyllaceae, based on 14 core mitochondrial genes; and one covering a representative group of all fungi (James et al. 2006) based on a subset of five mitochondrial genes (*cox1*, *cox2*, *cox3*, *cob*, and *atp6*). We used RevTrans (Wernersson and Pedersen 2003) with default settings to align nucleotide sequences of each gene while maintaining codon structure. Alignments were concatenated and partitioned by PartitionFinder (Lanfear et al. 2012) according to first, second, and third codon position. We performed model selection and phylogenetic reconstruction using the IQ-TREE program with 5,000 bootstraps and otherwise default settings (Nguyen et al. 2015). For the fungal phylogeny, we included a topological constraint tree (supplementary data 6, Supplementary Material online) to resolve deep divergences conformant to James et al. (2006). We also constrained the Lyophyllaceae clade to conform to our 14 gene analysis, as the five genes contain less phylogenetic signal to accurately resolve these closely related species.

We also ran a MrBayes v3.2.6 analysis (Ronquist et al. 2012) through the Cipres web server (Miller et al. 2010) for the Lyophyllaceae phylogeny with the following parameter settings: 50 million generations, sample frequency 5,000, nst=mixed, four chains, and a burnin percentage of 35. We used a Gamma model of sequence evolution with invariant sites. The topology used in this article was derived from the IQTree Maximum Likelihood analysis and since there were no supported topological conflicts with the Bayesian tree, we include the posterior probability support values from the MrBayes analysis with the ML bootstraps in order to indicate nodal support. We calculated silent and nonsilent substitution rates of mitochondrial genes for the Lyophyllaceae genomes using the R function kaks (package seqinr), using *Tricholoma matsutake* as outgroup species.

Whole-genome alignment was performed using progressiveMauve v.20150226, (Darling et al. 2004). Species with an IR had one copy of the IR removed to facilitate alignment of duplicated regions.

### Detection of G-Quadruplex Sequence Motifs

We used G4Hunter (Bedrat et al. 2016) to scan mtDNA sequences for putative regions capable of G-quadruplex formation. We used strict and relaxed settings (respectively, *w* = 25, *s* = 1.7, and *w* = 25, *s* = 1.2) to account for the inherent uncertainty of in silico detection of G4DNA. Because G4Hunter occasionally reports sequences with significant overlap, we eliminated sequences that overlapped a previously reported sequence by >50% of their length. This was done to avoid counting the same potential G-quadruplex twice or more.

### GC Skew Analysis

To identify potential locations of replication origins, we created cumulative GC skew graphs (Grigoriev 1998) of our mtDNA assemblies. We used DAMBE v.6.4.29 (Xia and Xie 2001) with default settings to generate GC skew data and integrated the results to obtain the cumulative GC skew.

## Results

### Overview of Mitochondrial Genomes

We assembled the complete mitochondrial genome for 12 species belonging to the Lyophyllaceae: seven unnamed species of *Termitomyces* covering the known diversity of the genus with strains from the three known genera of fungus-growing termites *Macrotermes*, *Microtermes*, and *Odontotermes* (Aanen et al. 2002; van de Peppel J.J., M. Nieuwenhuis, D.K. Aanen, Z.W. De Beer unpublished data), *B. zonata*, *T.rancida*, *M. boudieri*, *Tricholomella constricta*, and *Ast.parasitica*. All genomes contained a core set of protein-coding genes (table 1) ubiquitous for fungal mitochondria: *atp6*, *atp8*, *atp9*, *cob*, *cox1*, *cox2*, *cox3*, *nad1*, *nad2*, *nad3*, *nad4*, *nad4L*, *nad5*, *nad6*, and *rps3*; as well as 16 s and 23 s ribosomal subunits, and around 24 tRNAs (not including duplicates, precise number varies per species). The mtDNA of *B. zonata*, *T. rancida*, and all *Termitomyces* specimens featured a large IR region and therefore duplication of all genes contained within, though the gene content of the IR varied from species to species. All species except *Ast. parasitica* had introns in one or more mitochondrial genes. In particular, *cox1* often featured several introns, most of which harbor LAGLIDADG or GIY-YIG endonucleases, a phenomenon commonly found in basidiomycete fungi (Lang et al. 2007).

**Table 1:** Overview of Mitochondrial Genomes of 12 Lyophyllaceae Species Assembled and Annotated for This Study

Strain	GenBank ID	mtDNA Size (bp)	IR Size (bp)	GC Content (%)	Introns									
					atp9	cob	cox1	cox2	nad1	nad2	nad4	nad5	rns	rnl
*Asterophora parasitica*	MH725791	43,328	0	31.7	0	0	0	0	0	0	0	0	0	2
*Tricholomella constricta*	MH725800	65,087	0	25.3	0	1	0	0	0	0	0	0	0	2
*Myochromella boudieri*	MH725793	99,774	0	27.9	0	3	9	2	2	0	0	1	0	4
*Blastosporella zonata*	MH725792	200,401	56,326	34.8	1	6	6	3	0	0	1	2	2	4
*Tephrocybe rancida*	MH725794	126,794	33,754	38.3	0	2	3	2	0	0	0	0	0	1
*Termitomyces* sp. Mi166	MH725795	239,317	71,805	37.8	1	4	15	1	1	1	0	0	2	8
*Termitomyces* sp. T159	MH725799	157,156	41,609	39.3	1	2	4	1	0	0	0	0	1	3
*Termitomyces* sp. T32	MH725797	131,333	35,619	40.9	0	0	5	2	0	0	0	0	0	1
*Termitomyces* sp. T13	MH725796	155,430	44,175	34.5	0	3	6	2	0	0	0	0	2	2
*Termitomyces* sp. DKA19	MH743217	105,724	4,463	31	1	2	8	2	0	0	0	0	0	1
*Termitomyces* sp. T123	MH725798	124,711	28,228	33.1	0	1	6	2	0	1	0	1	0	3
*Termitomyces* sp. T132	MG783568	146,712	37,211	31.3	1	1	8	3	0	0	0	0	0	2

We found four distinct types of autonomous linear mitochondrial plasmids, two in each of two *Termitomyces* strains (T132 and T123). Such plasmids are commonly found in fungal mitochondria, for example, maranhar/kalilo in *Neurospora* (Chan et al. 1991; Court and Bertrand 1992), and are capable of (partial) insertion within mtDNA. They were presumably assembled by IOGA due to partial shared homology of the plasmid sequence and regions of mtDNA that derive from plasmid insertions. We could distinguish autonomous plasmid sequences from inserted ones using several key features: autonomous plasmids were placed by the assembler in a separate contig whereas inserts were merged with other mtDNA sequences; autonomous plasmids were assembled completely including both terminal IRs (TIRs) and both intact DNA and RNA polymerases, while inserts were generally degenerate and had lost either the TIRs and/or the polymerases; autonomous plasmids showed different (∼2-fold higher) coverage compared with mtDNA; and finally, reads mapping to autonomous plasmids showed no paired reads mapping to mtDNA sequences.

When BLASTing plasmid sequences against our mtDNA assemblies, we found significant *E*-value (≪1e-20) matches with the host mtDNA, as well as occasional matches with mtDNA of other species, suggesting these plasmids were present in the common ancestor of other strains as well, and inserted parts of their DNA in the host mtDNA.

### Mitochondrial IR

We detected the mitochondrial IR in our assemblies by a 2-fold increase in read coverage in the duplicated region, due to the assembler merging the identical copies. The inverted nature of the duplication was suggested by inverted read pairings between the ends of the assembled sequence and the borders of the duplicated region. To confirm the presence of the IR, we designed primers around the predicted border regions of the IR and the single copy regions. We succeeded in amplifying products for all four border regions in two strains of *Termitomyces* (T13 and T132, supplementary data 2*C*, Supplementary Material online). We took this result as confirmation of the IR hypothesis in all samples with a similar coverage peak. We then copied and pasted the IR sequence in its inverted position to complete each genome sequence. We defined the single copy regions as single copy region 1 (SC1, which for all species barring *B. zonata* contains *cox1*) and single copy region 2 (SC2, which always contains *cox3* and *rps3* at its edges).

The gene content of the IR varies somewhat per species, but in most cases it encompasses the small and large ribosomal subunits, *atp6*, *nad2*, *nad3*, *nad4*, *cox2*, and several tRNAs (table 2). The border regions with SC2 appear relatively stable in terms of gene content, with *cox3* and *rps3* always just on the outside of the IR. In contrast, border regions surrounding SC1 are highly variable. In *Termitomyces* sp. T132, the 23 s ribosomal subunit overlaps with the IR border, resulting in an incomplete copy of the subunit on one side of the IR. In *T. rancida*, *nad4* is partially duplicated in the same vein.

**Table 2:** Gene Content of Inverted Repeat Regions in mtDNA of *Termitomyces* and Related Fungi

Species	Genes in IR							tRNAs in IR									Genes on Border SC1	Genes on Border SC2
	rnl	rns	cox2	nad4	nad2	nad3	atp6	Ile	Val	Ala	Phe	Met	Thr	His	Met (2)	Met (3)		
*Blastosporella zonata*	x	x	x		x	x	x		x	x	x	x			x		tRNA-His/nad4	cox3/rps3
*Tephrocybe rancida*	x	x	x	x^a^	x	x	x		x	x	x	x	x	x	x	x	tRNA-Met(4)/nad4^a^	cox3/rps3
*Termitomyces* sp. Mi166	x	x	x		x	x	x	x	x	x	x	x	x	x	x		tRNA-Met(3)/nad4	cox3/rps3
*Termitomyces* sp. T159	x	x	x	x	x^a^		x		x	x	x	x	x	x	x		nad2^a^/cox1	cox3/rps3
*Termitomyces* sp. T32	x	x	x				x		x	x	x	x	x	x	x		nad4/cox1	cox3/rps3
*Termitomyces* sp. T13	x	x	x	x	x^a^		x		x	x	x	x	x	x	x		nad2^a^/tRNA-Gly	cox3/rps3
*Termitomyces* sp. DKA19				x^a^									x	x			tRNA-Ser/nad4^a^	cox3/rps3
*Termitomyces* sp. T123	x^a^	x	x	x	x	x	x	x	x	x	x	x	x	x			cox1/rnl^a^	cox3/rps3
*Termitomyces* sp. T132	x^a^	x	x	x	x	x	x	x	x	x	x	x	x	x			cox1/rnl^a^	cox3/rps3

Note.—Flanking genes for each set of border regions (IR-SC1, IR-SC2) are also shown in this table.

a Genes partially overlapping with IRs.

We compared silent substitution rate estimates of genes of the 12 mitochondrial genomes we sequenced in this study, to test whether genes inside the IR had lowered substitution rates. We found no systematic difference in substitution rates for IR-contained genes between species with and without an IR (supplementary data 3, Supplementary Material online). *K*_a_/*K*_s_ estimates were all <1 which suggests that all genes are under purifying selection as is typical for mtDNA (Soares et al. 2013).

To see if the IR harbored the replication origins as was found for *C. albicans* (Gerhold et al. 2010), we analyzed the cumulative GC skew profile for each mitochondrial genome with an IR (supplementary data 5, Supplementary Material online). We were unable to conclusively determine the locations of origins of replications from these graphs, as there was too much variation between profiles, presumably due to inversions and other rearrangements.

### Phylogenetic Analysis/Mauve

**Fig. 1.**
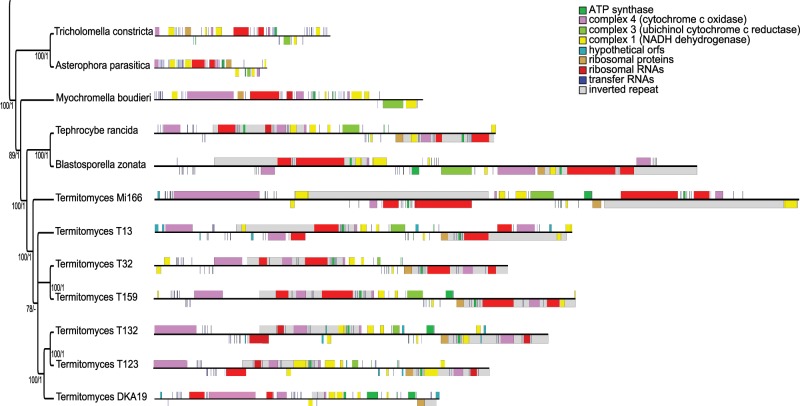
—Mitochondrial genomes of 12 Lyophyllaceae species. The phylogeny is rooted with *Tricholoma matsutake*, but the genome of this species is not included in the figure. Images of genomes created with OGDRAW (Lohse et al. 2013).

Our phylogenetic analysis (fig. 1) shows strong concordance with previous research; the monophyly of *Termitomyces* (Aanen et al. 2002; Frøslev et al. 2003), *B. zonata* and *T. rancida* as the sister clade, the position of *M. boudieri* outside the *T. rancida* clade and the monophyly of *Ast. parasitica* and *T. constricta* (Hofstetter et al. 2014; Bellanger et al. 2015). Our phylogenetic reconstruction shows that the IR has a single origin probably in the common ancestor of the *Termitomyces* clade and its sister clade containing *T. rancida* and *B. zonata*. *Myochromella boudieri* is currently the most closely related taxon to these clades without the IR. We did not find any subsequent losses of the IR in this study, although the span of the IR has significantly decreased in *Termitomyces* sp. DKA19. The three *Termitomyces* spp. associated with *Macrotermes* termites are monophyletic which is consistent with previous studies (Aanen et al. 2002; Nobre et al. 2011). The symbionts associated with *Microtermes* termites show paraphyly, with sp. Mi166 being early branching from the other *Termitomyces* species. Nobre et al. (2011) found a similar occurrence with one *Microtermes* symbiont being placed outside the main clade.

**Fig. 2.**
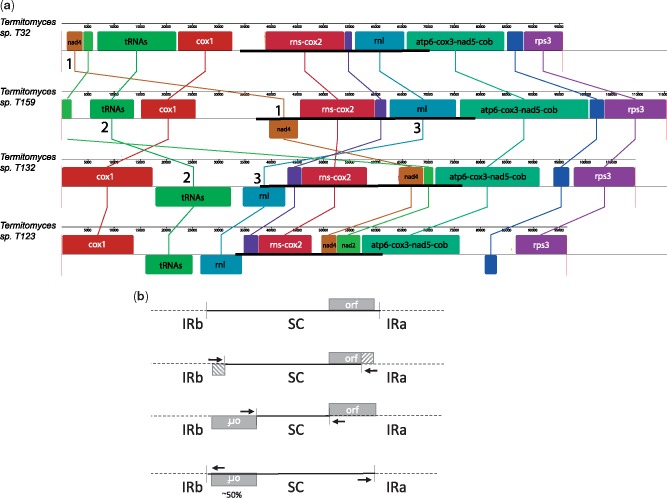
—(*A*) Mauve alignment of four *Termitomyces* mitochondrial genomes: sp. T32 (used as reference), T159, T132, and T123. Colored blocks indicate predicted homologies, which are connected through vertical lines. Genes associated with homologous regions are indicated in text for each block. If a block is shown underneath the line it indicates an inversion with respect to the reference. The inverted repeat is represented by a bold line for each genome (only one copy is shown). Several rearrangements are likely due to contraction/expansion of the inverted repeat: 1) The movement of *nad4* from an upstream position of *cox1* in SC1 of sp. T32 to the IR in the other species is probably a result of either contraction or expansion; 2) the repositioning of a large tRNA island from an upstream position of *cox1* in sp. T32 and T159 to a downstream position in sp. T132 and T123 most likely involved both contraction and expansion; 3) the large ribosomal subunit (rnl) is only partially included in the IR in sp. T132 and T123, showing a potential expulsion or enveloping in progress. (*B*) Example of gene translocation through successive inclusion and expulsion by the IR (Goulding et al. 1996). When the IR expands, for instance through illegitimate recombination, it can overlap a flanking ORF. This creates a copy of the overlapping part of the ORF on the opposite IR. The ORF may even end up completely within the IR, in which case two complete copies of the ORF are created. When the IR shrinks and expunges the ORF, one of the copies will disappear, resulting in an ∼50% chance of the ORF translocating to a new position.

Whole-genome alignment by Mauve (fig. 2*A*, only four species shown as representation) revealed multiple rearrangement events, including numerous rearrangements among *Termitomyces* species, several of which appear to be linked to contraction/expansion of the IR (fig. 2*B*). For example, the change of a number of tRNAs from an upstream position of *cox1* to a downstream position in several *Termitomyces* species probably occurred by successive inclusion and expulsion by the IR (Goulding et al. 1996).

### G4DNA

**Fig. 3.**
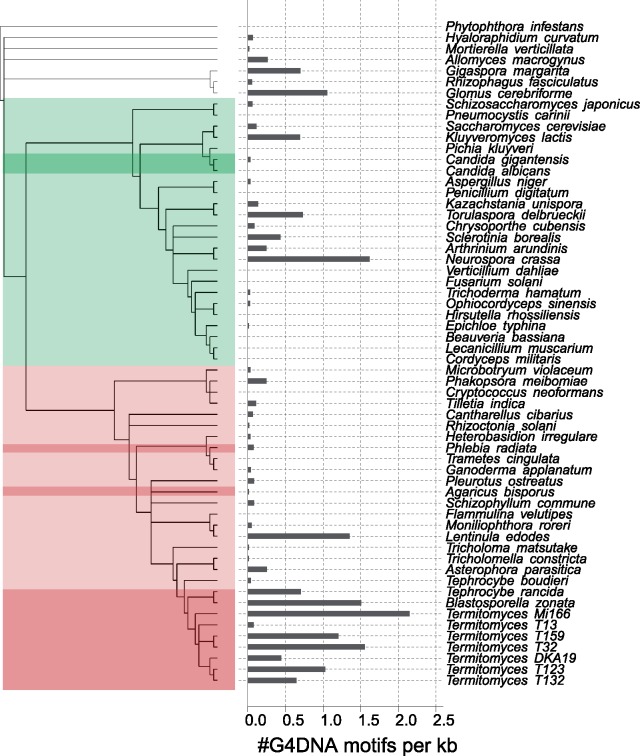
—Barplot showing average G4DNA motif content per 1-kb mtDNA for 62 species. Values were estimated with strict settings in G4Hunter (*w* = 25, *s* = 1.7). The phylogenetic tree was reconstructed using Maximum Likelihood with IQtree. Nodes with <70 bootstrap support were collapsed into polytomies. The tree was rooted with *Phytophthora infestans*. Ascomycetes are shaded in green and Basidiomycetes in red. Species with mitochondrial IRs are shaded in a darker hue.

We identified G4DNA motifs in most fungal mtDNA under both strict and relaxed settings of G4Hunter. The majority of fungi had comparatively low levels of G4DNA, but some species showed clear peaks of increased G4 content. Both strict and relaxed settings showed similar global patterns of G4DNA content, although for some species, the difference between the two settings was greater than for others (supplemental data 4, Supplementary Material online). This may partly be accounted for by a skewness in the false discovery rate, for instance due to differences in GC content or repetitiveness between genomes. Since the overall pattern of strict and relaxed settings was so similar, we will only discuss the results pertaining to the strict analysis here (fig. 3).

G4DNA motif content is a divergent character across fungi, with most species we analyzed showing relatively low frequencies of sequences with G4 potential (<0.5/kb), and several species showing distinctively high G4DNA content. These include the model species *Neurospora crassa*, as well as the basidiomycete *Lentinula edodes*. In addition, all but two *Termitomyces* species, as well as *B. zonata* and *T. rancida* all have estimates >0.5/kb.

**Fig. 4.**
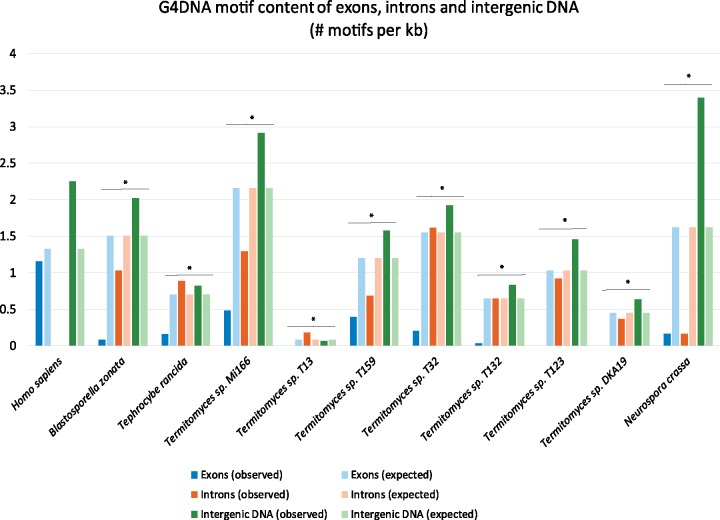
—Observed and expected G4DNA motif content for exons, introns, and intergenic regions in human and fungal mtDNA. Exons were only considered for the conserved protein-coding genes (*cox1-3, nad1-6, atp6, atp8, atp9, cob*, and *rps3*) and the ribosomal subunits (rns and rnl, while these are not protein coding they produce large, functional transcripts). Human mtDNA has no introns. For *Termitomyces* sp. DKA19, T13, and T123, no G4 motifs were observed in coding regions. Asterisks indicate significant deviation of observed values from the expected distribution (χ^2^ test, *P* < 0.05).

The high G4DNA motif content found in our mtDNA assemblies is similar to the high content found in human mtDNA (Bedrat et al. 2016). However, humans like any other vertebrate have miniature, gene-dense mtDNA molecules, with no introns and very little intergenic DNA. In contrast, fungal mtDNA often features numerous introns and long stretches of intergenic DNA. We compared G4DNA motif content of exons, introns, and intergenic DNA of human mtDNA and our fungal mtDNA assemblies, as well as *N. crassa*, to see if G4DNA was located differentially in these regions between species (fig. 4). We compared observed to expected values assuming unbiased distribution of G4DNA. We found that G4DNA motifs in fungi occurred significantly less in exons of conserved protein-coding genes (*cox1-3, cob, nad1-6, atp6, atp8, atp9*, and *rps3*), as well as the ribosomal subunits, than expected by chance (*P* < 0.05), whereas in human mtDNA, there was no bias.

**Fig. 5.**
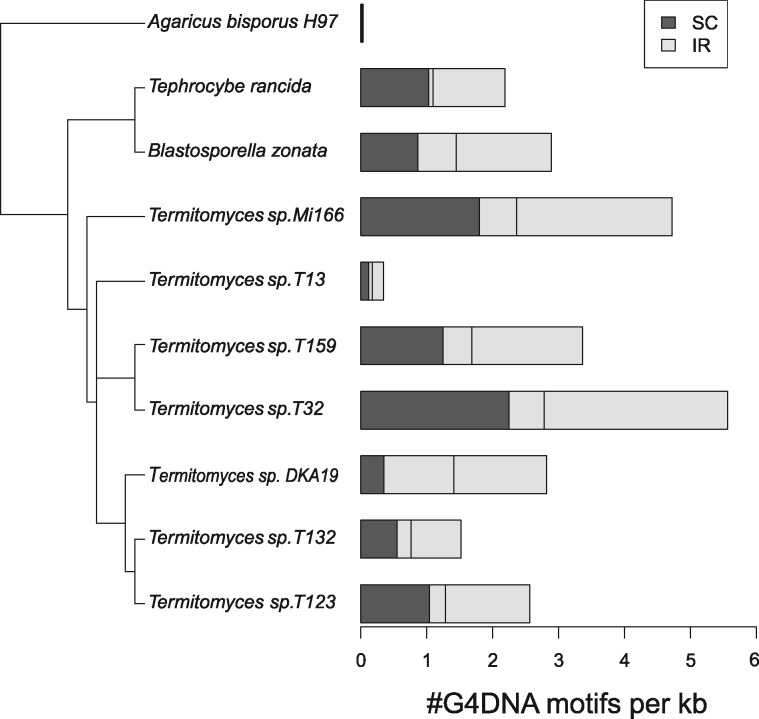
—Average G4DNA motif content per 1-kb mtDNA for Lyophyllaceae species with IRs, and *Agaricus bisporus* as outgroup. Dark gray denotes the G4DNA motif content for the SC region. Light gray indicates the G4DNA motif content of the IR. The expected 50/50 divide if the G4DNA motif content was equal for IR and SC is shown by a vertical line halfway along each bar. The phylogenetic tree is the same as that of figure 1, but rooted with *A. bisporus* rather than *Tricholoma matsutake* as it is the closest relative to the other species that has a mitochondrial IR.

To look for a possible relationship between the span of the IR and the frequency of G4DNA motifs, we tested whether G4DNA motifs were more common within the IR than in the SC regions, and found a consistently higher content of G4DNA motifs inside the IR. However, for some species, this difference was very small (fig. 5). A phylogenetic paired *t*-test (phyl.pairedttest, R package “phytools”) showed the difference in G4DNA motif content between IR and SC was statistically significant across species (*P* = 0.02). To exclude the possibility that this increase in G4 is simply due to a higher concentration of noncoding DNA in the IR, we calculated what percentage of DNA in the IR and SC regions was part of exons of conserved protein-coding genes or the ribosomal subunits. We found that in all but two cases, the percentage of exonic DNA was in fact higher in the IR than in the SC regions (table 3).

**Table 3:** Percentage of DNA in the IR and SC Regions That Belongs to Exons of Conserved Protein-Coding Genes (*cox1-3, cob, nad1-6, atp6, atp8, atp9* and *rps3*) and the Two Ribosomal Subunits, rns and rnl.

Species	Exons (%)	
	SC	IR
*Blastosporella zonata*	13.64	**15.50**
*Tephrocybe rancida*	18.91	**30.27**
*Termitomyces* sp. Mi166	13.20	**14.74**
*Termitomyces* sp. T13	15.52	**18.77**
*Termitomyces* sp. T159	14.37	**21.74**
*Termitomyces* sp. T32	**26.04**	24.85
*Termitomyces* sp. T123	18.91	**30.56**
*Termitomyces* sp. T132	16.58	**20.74**
*Termitomyces* sp. DKA19	**21.16**	5.18

Note.—Highest value for each genome is shown in bold.

## Discussion

The mitochondrial genomes of *Termitomyces*, *T. rancida*, and *B. zonata* are characterized by a large IR and an enrichment of G4DNA motifs. Although the co-occurrence of these two phenomena could be a neutral effect of hitchhiking, it is worth considering whether a functional relation between acquisition of the IR and proliferation of G4DNA exists.

Most G4DNA motifs are located in intergenic regions, among tRNA clusters and surrounding the rRNA subunits. Previous studies on nuclear G4DNA motifs have shown that G4DNA is more often located in promotor regions than expected by chance (Du et al. 2007; Yadav et al. 2008), and therefore may play a regulatory role. Some G4DNA motifs are situated in introns, seemingly embedded in the endonuclease genes. Insertion of GC clusters is a potential method of neutralizing endonuclease activity through frame shifts (Peris et al. 2017). However, most of the intronic ORFs we found remain intact despite the G4DNA. In some species, specifically *N. crassa*, *Termitomyces* sp. Mi166, and *Termitomyces* sp. T159, G4DNA occurs significantly less in introns than expected by chance (fig. 4).

Coding regions in fungal mtDNA almost never appear to contain G4DNA motifs (fig. 4), which suggests that embedding of G4DNA in coding sequences is selected against. This is in contrast to human mtDNA, where G4DNA motifs are found in coding regions close to the expected frequency given an unbiased distribution. A possible explanation for this is that humans, and likely vertebrates in general, may have more proteins capable of suppressing G4DNA formation in coding regions. A recent study suggests that vertebrates acquired a mechanism to remove G4-prone RNA transcripts (Pietras et al. 2018), possibly reducing the negative posttranscriptional effects of G4DNA motifs in coding regions. Our finding that fungi appear to have strong selection against G4DNA in coding regions suggests they lack such a mechanism.

Generally, G4DNA motif content of fungal mtDNA appears to be low (<0.5/kb) or even zero (fig. 3), but there are some notable exceptions to this trend. Fungi showing high frequencies of G4DNA motifs include the fungus *N. crassa*, a model for genetic and evolutionary studies. The only fungus for which mitochondrial G4DNA motif content was previously reported is *S. cerevisiae* (Capra et al. 2010), and from our analysis, this species appears to have a slightly enriched mitochondrial G4DNA motif content compared with the fungi we currently have data for. Whether G4DNA is a functional part of the genome or accumulates in a neutral fashion, our finding that some species completely lack G4DNA motifs in their mtDNA while in others it is an abundant feature suggests that in both cases mitochondria can function. That G4DNA can have an adverse effect on fitness is clear from its consistent absence from coding regions (fig. 4). As such it seems most likely that in those species where G4DNA is wholly absent selection against G4DNA also affects noncoding regions, or there is limited noncoding space for G4DNA to settle.

Previous studies have reported GC-rich motifs in fungal mtDNA, for example, those surrounding recombination sites in some yeasts (Dieckmann and Gandy 1987; Liachko et al. 2014), double-hairpin elements (DHEs) in *Allomyces macrogynes* and others (Paquin et al. 2000), and palindromic motifs found in *Neurospora* (Yin et al. 1981)*.* Although not all GC-rich motifs are capable of G-quadruplex formation, we have examined whether some of these sequences qualified as G4DNA motifs in our analysis. Of the 89 GC-rich DHEs reported for *All. macrogynes*, only four overlapped G4DNA motifs. Similarly, more than a hundred GC-rich clusters are reported for the mtDNA of the yeast *S. cerevisiae* (Wolters et al. 2015), while the estimated number of G4DNA motifs was eight. It therefore seems that most of these GC-rich motifs are unlikely to form G4DNA. However, in the case of *N. crassa*, the GC-rich elements reported in Yin et al. generally seem to overlap with G4DNA motifs. That they were not identified as such at the time is understandable considering G4DNA was still relatively obscure and its biological relevance unknown.

Large (1 kb+) mitochondrial IRs, while rare in fungi, occur among others in *Candida*, *Agaricus*, *Phlebia radiata*, and *Agrocybe aegerita*. The IRs found in *Termitomyces*, *T.rancida*, and *B. zonata* are unprecedented in terms of span, taking up half of the genome in most species. While this could be the result of a neutral process of incremental expansion of the IR, the co-occurrence of large amounts of G4DNA motifs provides a possible selective benefit for this increase in size. G4DNA is known to cause frequent DSBs, while IRs can efficiently repair DSBs through HR (Lambert et al. 2010). Consistent with this, G4DNA motifs are slightly but significantly more frequent within the IR than outside of it (fig. 5), which could indicate that selection against G4DNA is weakened in the IR because the deleterious effects of DSBs are mitigated. Although the difference between G4DNA motif content within and outside the IR is only marginal for some species, it is consistent across species. It should be noted that HR can occur outside the IR as well, between different copies of mtDNA. However, the IR can undergo HR even when other copies of mtDNA are absent, and the potential frequency of HR increases with repeat copy number (Fujitani et al. 1995).

The enrichment of G4DNA in the IRs of *Termitomyces* could also be due to the colocalization of replication origins in the repeats. The association of G4DNA with replication origins is supported in other organisms (Paeschke et al. 2011; Valton et al. 2014; Valton and Prioleau 2016), and the potential role of mitochondrial IRs in recombination-driven replication of mtDNA is supported by experimental results obtained from *C. albicans* (Gerhold et al. 2010). In chloroplasts of maize, the IRs function as termini of linear monomers of ptDNA, and harbor the replication origins (Oldenburg and Bendich 2016). Some studies (Grigoriev 1998; Gerhold et al. 2010; Xia 2012) have employed GC-skew analyses to estimate replication origins in mtDNA sequences, however, the reliability of this method is dependent on the replication method and is severely reduced if the genome is frequently rearranged. We produced GC-skew graphs for our assembled mtDNA sequences but could not conclusively determine replication origin positions from them (supplementary data 5, Supplementary Material online).The IR itself appears to show no reduced mutation rate in contrast to what was observed in some other studies (Palmer and Thompson 1982; Maier et al. 1995) (supplementary data 3, Supplementary Material online). However, HR is evident from the lack of divergence between the two copies of the repeat. Several key genes are duplicated by the IR, but it is unclear whether a dosage effect plays a role in the emergence of organellar IRs, since the role of mitochondrial genes is highly conserved and most species do not have IRs or duplicated genes in their mtDNA.

The IR is unstable between species in terms of span (table 1). The border regions show distinct behaviors: one side (IR-SC1) is very dynamic, and frequent gene rearrangements around this border result from regular expansions and retractions of the IR. Plasmid insertions surrounding the IR-SC1 border region may also indicate instability. However, the IR-SC2 border appears to be highly stable, with *cox3* and *rps3* always flanking this side of the IR (table 2). Significant gene rearrangements that probably involved the IR (fig. 2) suggest the gene content of the IR was more variable in the ancestors of some species.

*Termitomyces* sp. DKA19 is peculiar due to the greatly reduced size of its IR (table 1). Despite its reduced size the IR is centered on a pocket of locally enriched G4DNA motifs, giving more credence to the hypothesis that the IR is in some way affecting the G4DNA content. However, it remains to be seen whether the decrease in IR span caused a reduction in global G4DNA content or was rather a result from that reduction. Conversely, in *Termitomyces* sp. T13, the number of G4DNA motifs is highly reduced across the entire length of the genome, but the size of the IR has been maintained.

Organellar IRs, including those of plant chloroplasts, stramenopile mtDNA, and all but one reported in this study (*Termitomyces* sp. DKA19), often encompass the ribosomal subunits. This mimics the multitudinous tendency of nuclear ribosomal DNA, and it may be selectively beneficial to harbor the highly conserved rDNA in a region of increased genomic stability. As ribosomal DNA may be associated with G4DNA (Capra et al. 2010), the increased G4DNA content of the IR of *Termitomyces* and relatives therefore could simply be due to the presence of rDNA. However, that would not explain why the IR of *Termitomyces* sp. DKA19, which does not contain ribosomal DNA, has an elevated G4DNA motif content compared with the rest of the genome.

It is unclear how the IR originated. A small mitochondrial IR in *Agr.aegerita* originated as the result of an insertion of a mitochondrial plasmid. Such plasmids and their insertion sites were also detected in several of our genome reconstructions. The insertion mechanism of these linear plasmids involves an expansion of their TIRs, and such an event may have given rise to the progenitor of the large IR observed in *Termitomyces* and its sister clade.

## Conclusion

We report a large IR and an apparent increase in G4DNA content in the mitochondrial genomes of *Termitomyces*, *B. zonata*, and *T. rancida*. We propose two nonmutually exclusive functional explanations for the observed correlation between the two structural phenomena: 1) the IR helps repair DSBs caused by G4DNA; 2) both structures are involved in the replication of mtDNA. We also provide the first comparative analysis of G4DNA content in mtDNA in fungi. The large discrepancy in mitochondrial G4DNA content between fungal species raises an opportunity for examining the effects of G4DNA on genome stability, replication, recombination, and transcription. Such studies would benefit from comparing similar species with different G4DNA content. In addition, some of the fungi with high mitochondrial G4DNA content are already established model species, *N. crassa* in particular. These species would be ideal candidates for experimental studies on G4DNA function and evolution, and could improve our insight in how G4DNA affects our own (mitochondrial) DNA.

Finally, we have shown that in fungal mtDNA coding regions are significantly depleted in G4DNA motifs, in contrast to humans. This suggests that fungi have stronger selection against G4DNA in exons, as they might lack the means to regulate G4DNA formation posttranscriptionally.

## Supplementary Material

Supplementary data are available at *Genome Biology and Evolution* online.

## Supplementary Material

Supplementary_Material_evz122

## Notes

**Data deposition:** Sequence data for this project has been deposited at NCBI GenBank under the accessions MG783568, MH725791-MH725800, and MH743217.
