# Supplementary material for: Enrichment of G4DNA and a Large Inverted Repeat Coincide in the Mitochondrial Genomes of Termitomyces
Source: Genome Biol Evol. 2019 Jun 18;11(7):1857–69. doi: 10.1093/gbe/evz122 (PMC6609731; doi:10.1093/gbe/evz122)
Supplement: Supplementary_Material_evz122 [file supplementary_material_evz122.zip › Supplementary_Data_1-2_DNA_isolation_protocol_for_Termitomyces.docx]

**Supplementary Data 1**

**DNA isolation protocol for *Termitomyces***

**Growing the material**

1. Harvest the mycelium by scraping off the material. Try to pick up the least amount of agar possible. Transfer the material to safe-lock, 1.5 ml Eppendorf tubes together with 4-5 glass pearls for the bead beater.

**Disrupting the cells and proteins**

2. Crush the material by freezing with liquid N2 and using the bead beater for 10 seconds.

3. Repeat step 2.

 If the material does not appear to be disrupted enough (e.g. if large clumps of tissue are still present)

repeat the procedure.

4. Add 500 μl CTAB (2% Hexadecyl.trimethyl.ammoniumbromide, 1.4M NaCl, 20mM EDTA, 100mM

Tris.HCl pH=8) and 2 μl Proteinase K (15-20 mg/ml)

5. Put the Eppendorf tubes in the shaker and incubate them for 1 hour at 65 °C.

 Alternatively incubate them overnight at 55 °C

**Phase separation - From here on work in the fumehood!**

6. Add 500 μl Chloroform : Isoamylalcohol (24:1) and mix gently by inversion.

7. Centrifuge for 15 min at maximum speed and room temperature (still in the fumehood!).

 If the chloroform and water phase are not separated well enough centrifuge

longer.

8. Carefully pipet off the water phase (top phase!) and transfer to a new Eppendorf tube. Do not

be greedy; better to transfer less DNA then take some of the chloroform phase. Discard the

lower (chloroform) phase to the proper waste container.

**DNA precipitation**

9. Add an equal volume (~300-400 μl) of ice-cold Isopropanol.

10. Put the tubes at -20 °C for a minimum of 20 minutes.

11. Centrifuge at maximum speed for 15 minutes at 4 °C

12. Remove the fluid from the pellet.

**Washing DNA – from here on you can stop working in the fumehood**

13. Add 300 μl 100% Ethanol to the pellet

14. Centrifuge at maximum speed for 5 minutes at room temperature

15. Remove the fluid from the pellet

16. Add 300 μl 70% Ethanol to the pellet

17. Centrifuge at maximum speed for 5 minutes at room temperature

18. Remove the fluid from the pellet

19. Dry the pellet in a vacuum for a couple of minutes

**Collect DNA**

20. Dissolve the dry pellet in 50 μl MQ water and store at -20 °C

**Supplementary Data 2**

Primers

PCR reaction mixture

5x GoTaq buffer 5 μl

25mM MgCl2 2 μl

10mM dNTP 1 μl

Forward primer 1 μl

Reverse primer 1 μl

GoTaq 0.1 μl

Template DNA 1:10 1 μl

MQ water 13.9 μl

Touchdown PCR program

Initial denaturation at **94⁰C** for **5min**

**20** cycles (**60s** at **94⁰C**; **60s** at **60⁰C**; **60s** at **72⁰C**, decreasing annealing temperature by **0.5⁰C** after each cycle)

**20** cycles (**60s** at **94⁰C**; **60s** at **55⁰C**; **60s** at **72⁰C**)

Final elongation at **72⁰C** for **10 min**

|  | Strain | Forward primer | Reverse primer |
| --- | --- | --- | --- |
| A | T132 | TACCGCCATTGAACTCTAC | TTACGCGTGCAGATGCGCGT |
| B | T132 | TTACGCGTGCAGATGCGCGT | AGTTGGTGATCTCCTTGAAG |
| C | T132 | GGGGTTATTAATCACTGGAT | ACTTCTTTGGTATGGTGACC |
| D | T132 | TTAAGGCCCTCCTTTCTGAT | ATCCAGTGATTAATAACCCC |
| H | T132 | CACAGCTGAGTTAAGTCCTG | GGGGTTATTAATCACTGGAT |
| E | T13 | GGGGTTATTAATCACTGGAT | TATGATTGCCTCCTACGGAG |
| F | T13 | TATGATTGCCTCCTACGGAG | CCTTCTGTTATGACATCTCT |
| G | T13 | CCGTACTTCGTACTTCGTAC | GCTTTAGTGGAAGACTCCGA |
| I | T13 | CGAGATAGAGATTTCTAGGC | CCGTACTTCGTACTTCGTAC |

Gel pictures:


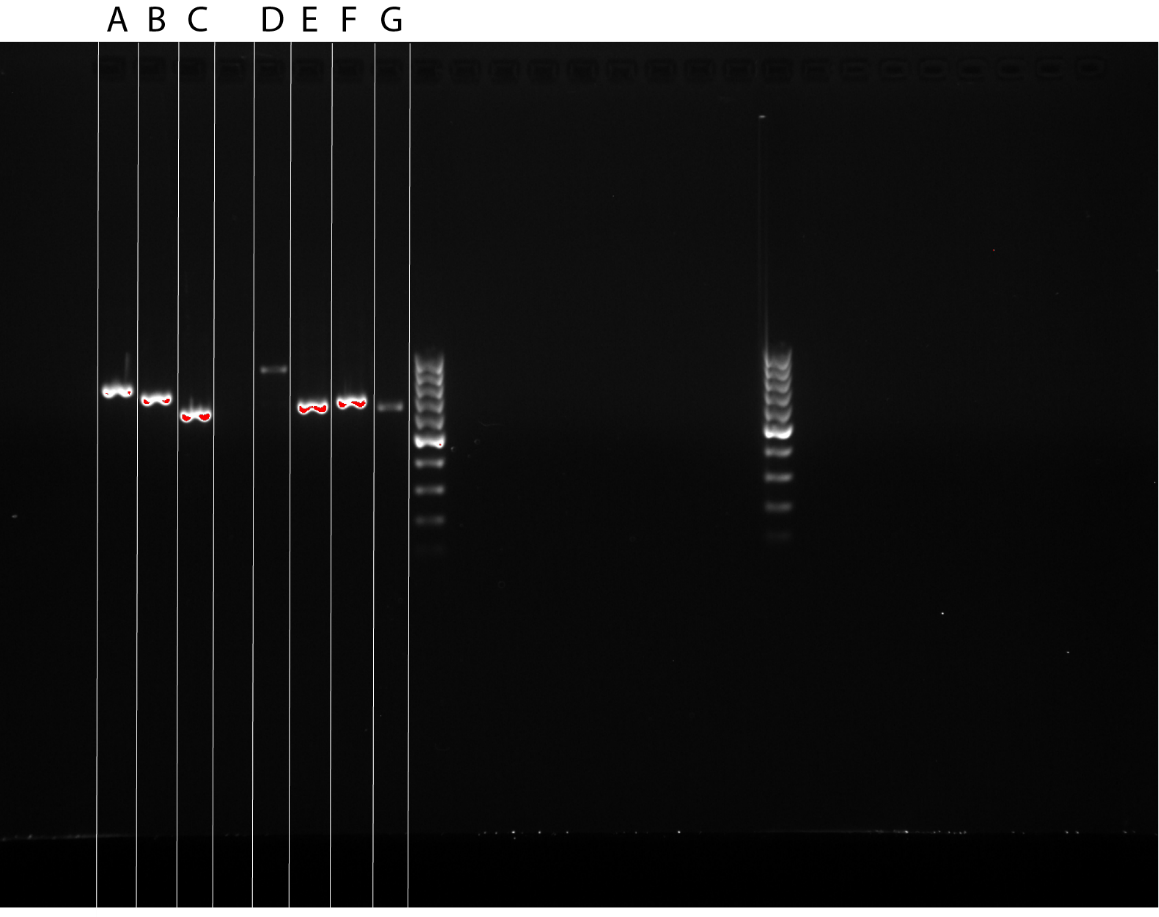

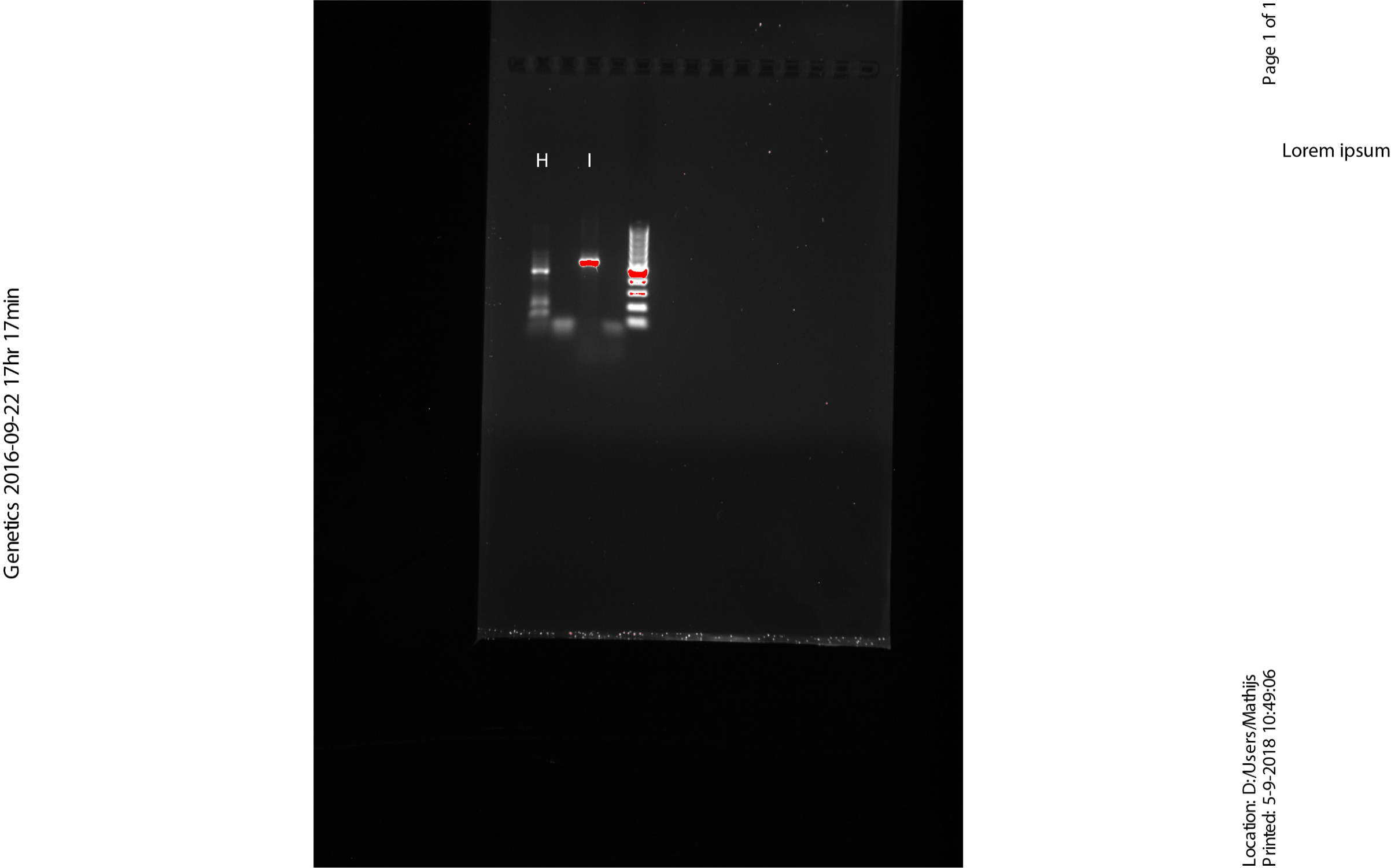


Figure 1 – PCR products for all nine primer combinations. Lanes A,B,C,D and H correspond to the four IR/SC border regions in *Termitomyces* sp. T132. One border region was tested with two primer combinations to increase the chance of success as the sequence was predicted to cause problems for amplification. Lanes E,F,G and I correspond to the four border regions in *Termitomyces* sp. T13. All reactions yielded a product, confirming the presence of an inverted repeat in two locations in each mitochondrial genome.
