# Supplementary material for: Enrichment of G4DNA and a Large Inverted Repeat Coincide in the Mitochondrial Genomes of Termitomyces
Source: Genome Biol Evol. 2019 Jun 18;11(7):1857–69. doi: 10.1093/gbe/evz122 (PMC6609731; doi:10.1093/gbe/evz122)
Supplement: Supplementary_Material_evz122 [file supplementary_material_evz122.zip › Supplementary_Data_3_Mutation_rates.docx]

**Supplementary Data 3**

**Mutation rates –Ka/Ks plots**

**
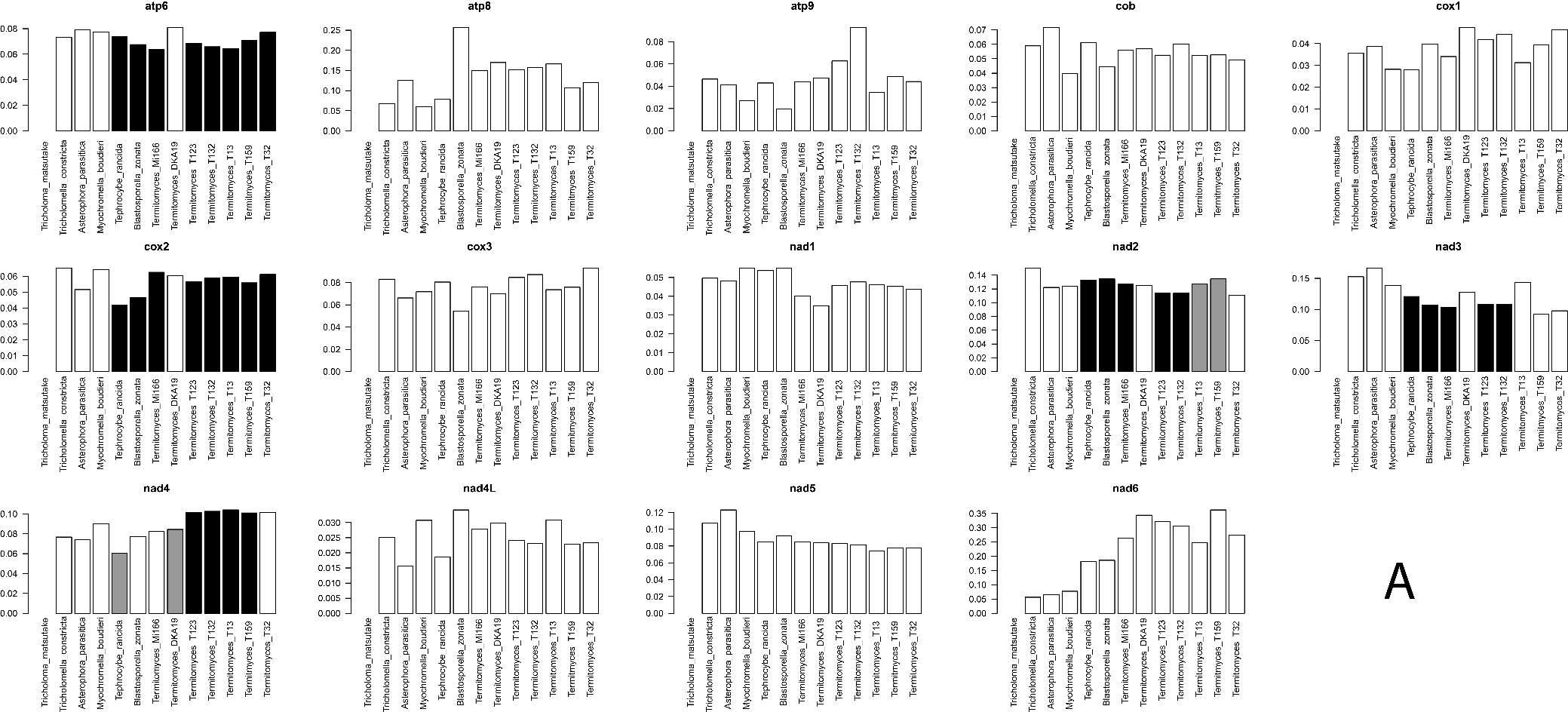
**

**
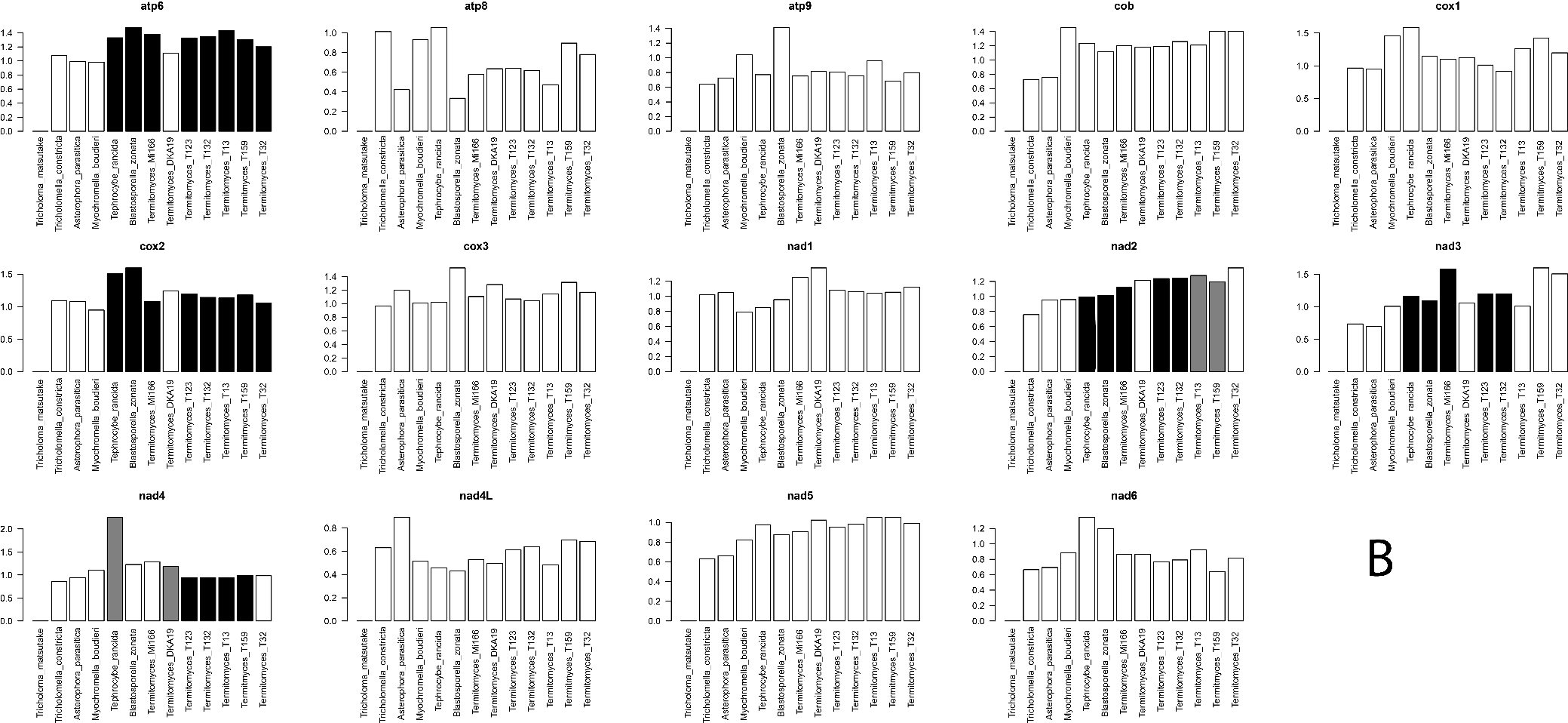
**

Figure 1 – Bar charts showing Ka/Ks ratios (A) and Ks estimates (B) for each mitochondrial gene for each species sequenced for this study, with *T. matsutake* as outgroup. Genes contained within the mitochondrial inverted repeat are denoted by black bars, while genes partially within the IR are shown with grey bars. All genes appear to be subject to purifying selection (Ka/Ks < 1). Genes (partially) within the IR do not appear to have systematically higher or lower synonymous substitution rates than genes outside of it.
