# Supplementary material for: Enrichment of G4DNA and a Large Inverted Repeat Coincide in the Mitochondrial Genomes of Termitomyces
Source: Genome Biol Evol. 2019 Jun 18;11(7):1857–69. doi: 10.1093/gbe/evz122 (PMC6609731; doi:10.1093/gbe/evz122)
Supplement: Supplementary_Material_evz122 [file supplementary_material_evz122.zip › Supplementary_Data_5_CGC_skews.pdf]

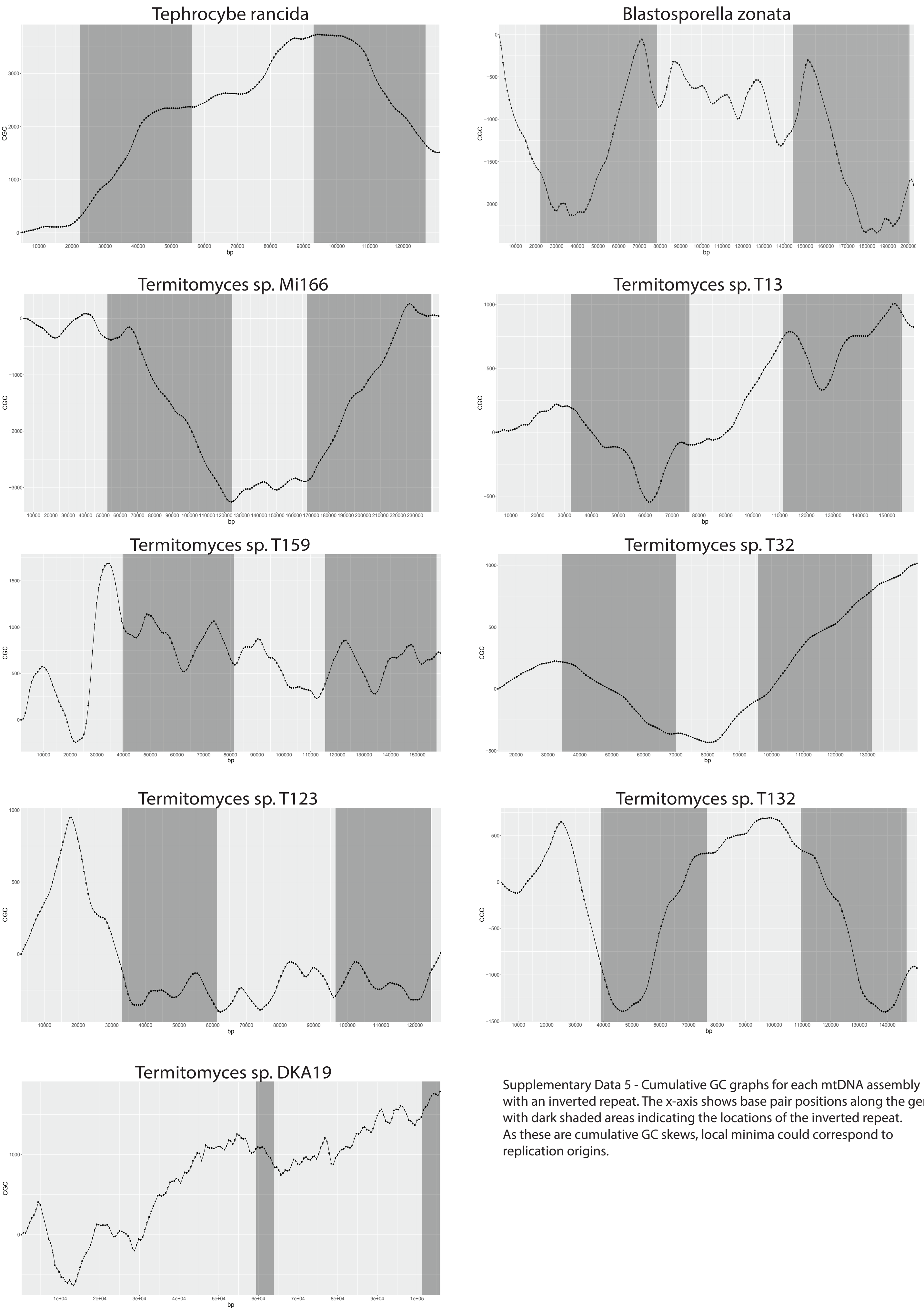

Supplementary Data 5 - Cumulative GC graphs for each mtDNA assembly with an inverted repeat. The x-axis shows base pair positions along the genome, with dark shaded areas indicating the locations of the inverted repeat. As these are cumulative GC skews, local minima could correspond to replication origins.
